# Phenotypic Switching and Filamentation in Candida haemulonii, an Emerging Opportunistic Pathogen of Humans

**DOI:** 10.1128/Spectrum.00779-21

**Published:** 2021-12-08

**Authors:** Yuchen Deng, Shuaihu Li, Jian Bing, Wanqing Liao, Li Tao

**Affiliations:** a Shanghai Key Laboratory of Molecular Medical Mycology, Department of Dermatology, Second Affiliated Hospital of Naval Medical University, Shanghai, China; b State Key Laboratory of Genetic Engineering, School of Life Sciences, Fudan University, Shanghai, China; The Ohio State University

**Keywords:** *Candida haemulonii*, phenotypic switching system, filamentous growth, glycerol, temperature dependent

## Abstract

Phenotypic plasticity is a common strategy adopted by fungal pathogens to adapt to diverse host environments. Candida haemulonii is an emerging multidrug-resistant human pathogen that is closely related to Candida auris. Until recently, it was assumed that C. haemulonii is incapable of phenotypic switching or filamentous growth. In this study, we report the identification of three distinct phenotypes in C. haemulonii: white, pink, and filament. The white and pink phenotypes differ in cellular size, colony morphology, and coloration on phloxine B- or CuSO_4_-containing agar. Switching between the white and pink cell types is heritable and reversible and is referred to as “the primary switching system.” The additional switch phenotype, filament, has been identified and exhibits obviously filamentous morphology when grown on glycerol-containing medium. Several unique characteristics of the filamentous phenotype suggest that switching from or to this phenotype poses as a second yeast-filament switching system. The yeast-filament switch is nonheritable and temperature-dependent. Low temperatures favor the filamentous phenotype, whereas high temperatures promote filament-yeast transition. We further demonstrated that numerous aspects of the distinct cell types differ in numerous biological aspects, including their high temperature response, specific gene expression, CuSO_4_ tolerance, secreted aspartyl protease (SAP) activity, and virulence. Therefore, transition among the three phenotypes could enable C. haemulonii to rapidly adapt to, survive, and thrive in certain host niches, thereby contributing to its virulence.

**IMPORTANCE** The capacity to switch between distinct cell types, known as phenotypic switching, is a common strategy adopted by *Candida* species to adapt to diverse environments. Despite considerable studies on phenotypic plasticity of various *Candida* species, Candida haemulonii is considered to be incapable of phenotypic switching or filamentous growth. Here, we report and describe filamentation and three distinct phenotypes (white, pink, and filament) in C. haemulonii. The three cell types differ in cellular and colony appearance, gene expression profiles, CuSO_4_ tolerance, and virulence. C. haemulonii cells switch heritably and reversibly between white and pink cell types, which is referred to as the “primary switching system.” Switching between pink and filamentous phenotypes is nonheritable and temperature-dependent, representing a second switching system. As in other *Candida* species, switching among distinct morphological types may provide C. haemulonii with phenotypic plasticity for rapid responses to the changing host environment, and may contribute to its virulence.

## INTRODUCTION

*Candida* species are recognized as the most prominent commensals of human hosts and comprise a range of dominant fungal pathogens in immunocompromised hosts. Among them, Candida auris is an emerging multidrug-resistant pathogen that has caused significant concern due to its rapid outbreaks and high mortality rates (1–4). Candida haemulonii was clinically isolated for the first time in 1984 from the blood of a patient, and it is closely related to C. auris in the Metschnikowiaceae clade (5, 6). Because of the high similarities of the biochemical profiles and antifungal resistance properties between C. haemulonii and C. auris, C. auris is frequently misidentified as C. haemulonii (6–9). Therefore, exploring biological characteristics highlighting the differences between the two species is important for accurate and rapid identification.

C. haemulonii reportedly causes both superficial and systemic infections, such as chronic otitis media, vaginal candidiasis, peritonitis, and candidemia (10–13). Although regarded as a rare *Candida* species, it has been attracting considerable attention due to its multidrug resistance to amphotericin B (AMB), triazoles, and echinocandins (14–17). Switching among diverse morphological phenotypes, known as phenotypic plasticity, is a common strategy adopted by *Candida* species to adapt to diverse environments, respond to antifungal drugs, and cause infections (18, 19). However, until recently, it had been assumed that C. haemulonii cannot undergo phenotypic switching or filamentous growth.

The best example of a phenotypic switching system is yeast-filament transition and the white-opaque switch in Candida albicans (20, 21). Yeast-filament transition has been considered as a common response to environmental changes. A range of host environmental factors, such as elevated CO_2_ levels, neutral pH, physiological temperature (37°C), and the presence of *N*-acetylglucosamine, induce filament development. In contrast, acidic pH, low temperature, and rich nutritional conditions promote the stabilization of the yeast phenotype (20, 21). In the white-opaque switching system, C. albicans cells switch heritably and reversibly between the white and opaque cell types, which show differences in cellular morphology, mating, secreted aspartyl protease (SAP) activity, and virulence (22–25). To better adapt to the changing host environment, white and opaque cells could undergo filament development, showing an integration of the white-opaque switching and yeast-filament transition systems in C. albicans (20, 26). This integrated phenotypic system has been widely considered to lead to increased complexity and plasticity of phenotypic switching, as well as enhanced environmental adaptation ability. Recently, a novel phenotypic switching system that contains both a heritable and a nonheritable switch has been demonstrated in C. auris (27). The heritable switch represents transitions between the typical yeast and filamentation-competent (FC) yeast/filamentous phenotypes, which is triggered by passage through a mammalian host. The nonheritable switch involved in the transition between the FC yeast and filamentous phenotype is demonstrated to be temperature-dependent.

The morphological diversity and plasticity of *Candida* species are strongly associated with their abilities to adapt to ever-changing environments and cause infection (18, 19). White cells are more virulent than opaque cells in systemic infections, while opaque cells are better at colonizing cutaneous tissues (24, 25). Filamentous cells are more invasive and penetrate host tissues more effectively than yeast form cells. The ability to undergo filamentation is a defining feature of virulence across *Candida* species (28).

In this study, we report and describe two novel cell types (pink and filament) and two phenotypic switching systems, a primary white-pink switch and a secondary yeast-filament switch, in C. haemulonii. Numerous biological aspects differ between the white, pink, and filament cell types, including cellular/colonial morphology, specific gene expression, CuSO_4_ tolerance, SAP activity, and virulence. Cells switch reversibly and heritably between the white and pink phenotypes. The presence of glycerol or low temperatures favor the filamentous phenotype, whereas high temperatures promote the yeast phenotype. Furthermore, global gene profiles and virulence assays suggest that various features of distinct phenotypes provide C. haemulonii with an advantage in the ever-changing host environment.

## RESULTS

### Discovery of the pink phenotype and the white-pink switching system in *C. haemulonii*.

**FIG 1:**
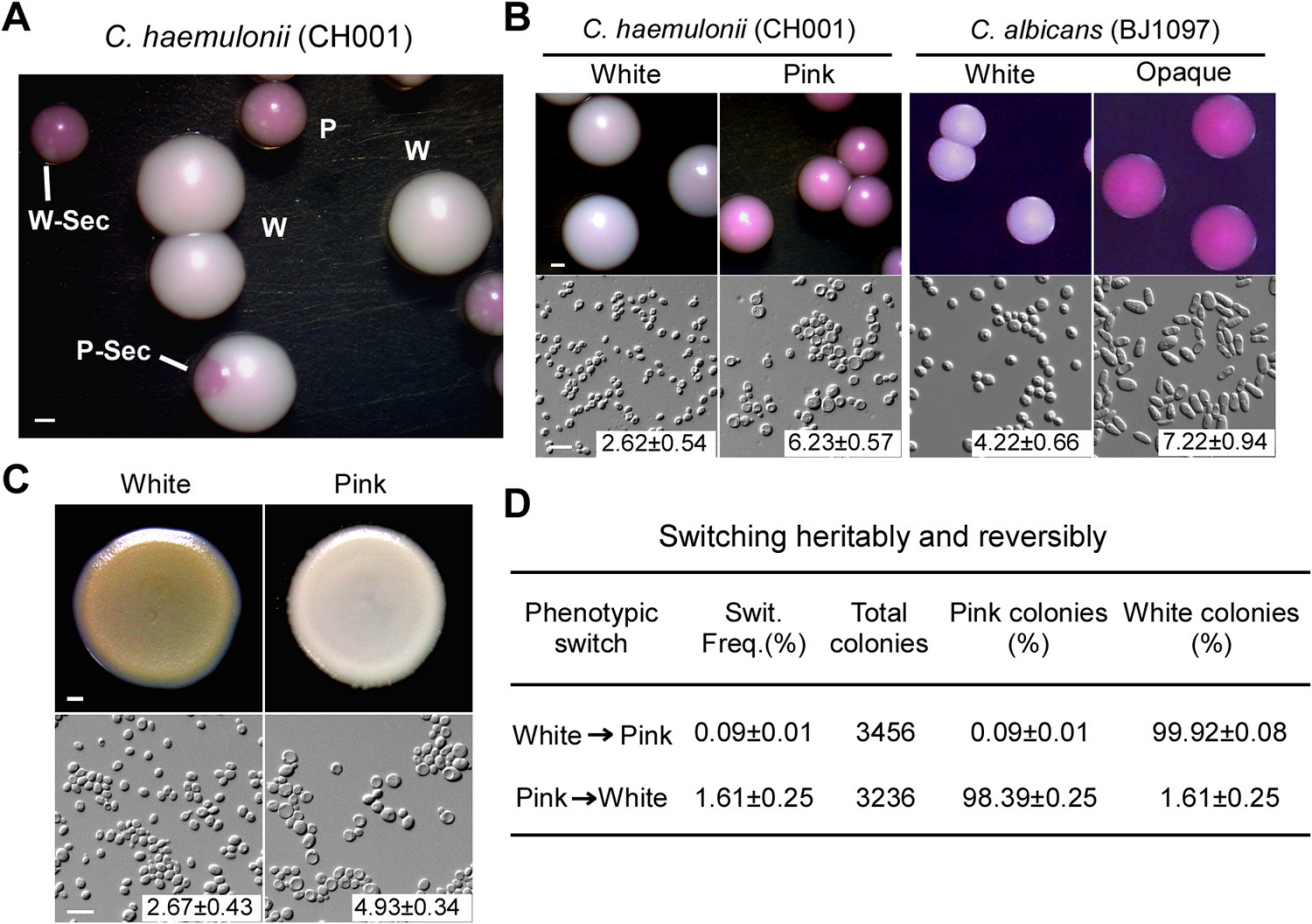
Pink phenotype and the white-pink switching system of C. haemulonii on YPD medium. (A) Morphologies of white, pink, and sectored colonies of C. haemulonii on YPD agar containing phloxine B dye. The colonies were imaged after 5 days of growth at 25°C. The pink phenotype, but not the white phenotype, was stained pink with phloxine B. W, white; W-Sec, white sector; P, pink; P-Sec, pink sector. Scale bar, 1 mm. (B) Colony and cellular morphologies of distinct phenotypes of C. haemulonii (CH001) and C. albicans (BJ1097). White and pink cells of C. haemulonii were grown on YPD agar containing phloxine B for 5 days at 25°C. White and opaque cells of C. albicans were grown on Lee’s glucose medium containing phloxine B for 5 days at 25°C. The cellular length/diameter of each phenotype was indicated in the corresponding image. Scale bar for colonies, 1 mm; Scale bar for cells, 10 μm. (C) Morphologies of white and pink colonies of C. haemulonii on YPD agar containing 1 mM CuSO_4_. White and pink cells (1 × 10^7^ cells) in 10 μl double-distilled water were spotted onto YPD- CuSO_4_ medium plates and grown at 25°C for 2 days. The mean length/diameters of white and pink cells were indicated in the corresponding images. Scale bar for spots, 1 mm; Scale bar for cells, 10 μm. (D) Switching frequencies of the white-pink bistable switching system on YPD medium at 25°C. A single colony of the white or pink phenotype was replated onto YPD agar containing phloxine B. The total number of colonies is the sum of three experiments. Switching frequencies represent the average percentages of white or pink colonies grown on YPD agar. Strain CH001 was used.

We isolated the C. haemulonii strain CH001 from the blood of a patient at Changzheng Hospital, Second Military Medical University in Shanghai, China. Analysis of rDNA sequences of the 18S internal transcribed spacer (ITS) region verified that CH001 is indeed a C. haemulonii strain (Fig. S1 in the supplemental material). When this strain was grown on yeast-peptone-dextrose (YPD) agar plates containing the red dye phloxine B at 25°C (25), we observed two distinct colony phenotypes, including white and pink colonies (Fig. 1A). White colonies were similar to the colonies formed by standard laboratory strains of C. haemulonii and appeared large and white. Pink colonies were consistently smaller than white colonies after equal periods of incubation and could be stained pink on phloxine B-containing YPD medium. Both white and pink phenotypes produced round and budding yeast cells on YPD medium, but the pink cells exhibited two to three times the volume produced by white cells (Fig. 1B). Although both C. haemulonii pink cells and C. albicans opaque cells formed pink colonies on phloxine B-containing media, the cellular morphologies were highly different. Pink cells of C. haemulonii were round and relatively small, while opaque cells of C. albicans were elongated and large (Fig. 1B). Moreover, the effect of CuSO_4_ on the morphology of C. haemulonii was investigated. When cells from white and pink colonies on phloxine B-containing YPD were grown on CuSO_4_-containing agar medium, they exhibited opposite color intensities (dark brown and light brown, respectively, Fig. 1C). Subsequently, we revealed that C. haemulonii cells could switch heritably and reversibly between the white and the pink phenotypes. On YPD medium at 25°C, the switching frequencies of white-to-pink and pink-to-white were (0.09 ± 0.01)% and (1.61 ± 0.25)%, respectively, indicating a novel white-pink switching system of C. haemulonii (Fig. 1D). Considering that the switching frequencies between white-to-opaque and opaque-to-white in C. albicans (BJ1097 a/α) were also very low (<0.2) on YPD medium (29), we proposed that the distinct phenotypes of both C. haemulonii and C. albicans are relatively stable under this culture condition.

### The presence of the white-pink switching system in other clinical isolates of *C. haemulonii*.

To test whether other clinical strains of C. haemulonii could undergo white-pink switching, 20 C. haemulonii strains isolated from different hospitals in China were plated on YPD + phloxine B medium and cultured at 25°C. The results showed that 15 of them are competent at white-pink switching. Six examples are shown in Fig. S2 in the supplemental material. As expected, both the colony and cellular morphologies of white and pink cells were similar to those of strain CH001. Moreover, these strains were isolated from various clinical specimens including pus, venous catheter, and blood (15) (Table S1). Taken together, these results suggest that the white-pink switch is a general feature of clinical strains of C. haemulonii.

### White and pink cells differ in growth rate under physiological temperature.

**FIG 2:**
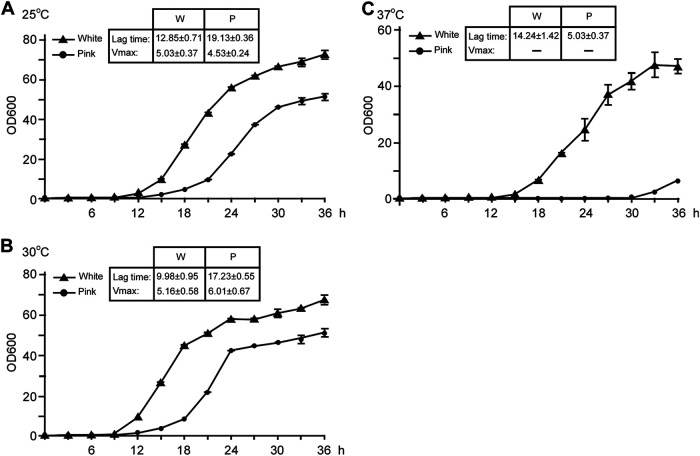
Growth curves of white and pink cells under different temperatures. Cells from white and pink colonies on YPD agar plates were inoculated into liquid YPD medium for growth overnight at 25°C. Cells were then washed twice with double-distilled water and reinoculated into liquid YPD medium at a concentration of 2 × 10^6^ cells/ml and incubated at 25°C (A), 30°C (B), and 37°C (C). The OD600 values were measured at different time points as indicated. Three biological repeats were performed. Growth curve fitting of viable count data was performed using DMFit program (https://browser.combase.cc/DMFit.aspx) to measure growth parameters using the Baranyi & Roberts model. The maximal growth rate (Vmax, OD/h) and lag time (h) were shown in each figure. W: white; P: pink; *x* axis, incubation time; *y* axis, OD600 value.

Temperature is an important environmental factor that efficiently regulates the morphology of *Candida* species (21). We further examined the effect of various temperatures on cell growth of the white and pink phenotypes. Cells of the two cell types were inoculated into liquid YPD medium and grown at 25, 30, and 37°C. The growth rate was determined by measuring the OD600 of each sample at different time points. As shown in Fig. 2, the pink cells exhibited a severe growth defect at physiological temperature (37°C), and they grew more slowly than the white cell type at both 25°C and 30°C. To verify these results, switching frequency between white and pink cells grown in liquid YPD medium was examined by counting CFU/ml (CFU per ml) counting method. As shown in Table 1, the switching frequency was constantly low during the whole culture period, thus confirming the reliability and validity of the growth rate analysis. Taken together, these results suggest that white and pink cells are relatively stable under laboratory culture conditions, and white cells are more resistant to physiological temperature than pink cells.

**TABLE 1:** Switching frequencies between white and pink phenotypes in liquid YPD medium^*a*^

Culture time	25^o^C				30^o^C				37^o^C			
	Total W colonies	Swit. freq	Total P colonies	Swit. freq	Total W colonies	Swit. freq	Total P colonies	Swit. freq	Total W colonies	Swit. freq	Total P colonies	Swit. freq
3 h	2,288	0.05 ± 0.09	1964	0.33 ± 0.34	3166	0.13 ± 0.06	2048	0.93 ± 0.16	1431	0.00 ± 0.00	0	–
6 h	3,135	0.03 ± 0.06	2443	0.40 ± 0.37	4084	0.03 ± 0.04	2799	0.49 ± 0.12	932	0.00 ± 0.00	0	–
9 h	4,208	0.08 ± 0.09	3731	0.60 ± 0.12	5116	0.02 ± 0.03	3308	0.50 ± 0.55	1525	0.00 ± 0.00	0	–
12 h	3,620	0.05 ± 0.05	4556	0.44 ± 0.06	3456	0.05 ± 0.09	2525	0.58 ± 0.28	2472	0.00 ± 0.00	0	–
15 h	4,520	0.08 ± 0.09	4483	0.74 ± 0.10	5868	0.02 ± 0.03	3264	1.39 ± 0.41	2497	0.00 ± 0.00	0	–
18 h	3,942	0.00 ± 0.00	2602	0.77 ± 0.31	5114	0.10 ± 0.03	2829	1.66 ± 0.38	1234	0.00 ± 0.00	0	–
21 h	5,970	0.02 ± 0.03	4260	1.30 ± 0.49	4923	0.02 ± 0.03	3545	2.37 ± 1.50	1870	0.00 ± 0.00	0	–
24 h	5,151	0.02 ± 0.03	2388	1.26 ± 0.50	4290	0.07 ± 0.07	2100	1.99 ± 1.03	1379	0.00 ± 0.00	0	–
27 h	5,132	0.02 ± 0.03	2513	2.19 ± 1.41	4130	0.00 ± 0.00	2888	2.14 ± 0.39	1245	0.00 ± 0.00	0	–
30 h	5,394	0.04 ± 0.03	4112	2.51 ± 1.27	4873	0.04 ± 0.03	4429	3.02 ± 0.52	992	0.00 ± 0.00	0	–
33 h	5,138	0.06 ± 0.06	3136	3.64 ± 1.20	4948	0.06 ± 0.06	3555	2.63 ± 1.01	944	0.00 ± 0.00	0	–
36 h	4,371	0.02 ± 0.03	3799	2.32 ± 0.91	5328	0.06 ± 0.06	2900	3.53 ± 0.86	924	0.00 ± 0.00	0	–

a W, white; P, pink; Swit. freq., Switching frequency (%). Switching frequencies represent the average percentages of white or pink colonies grown on YPD agar. “–” indicates that no cell growth was observed. This table is related to Fig. 2.

### Discovery of the filamentous phenotype and pink-filament switching system.

**FIG 3:**
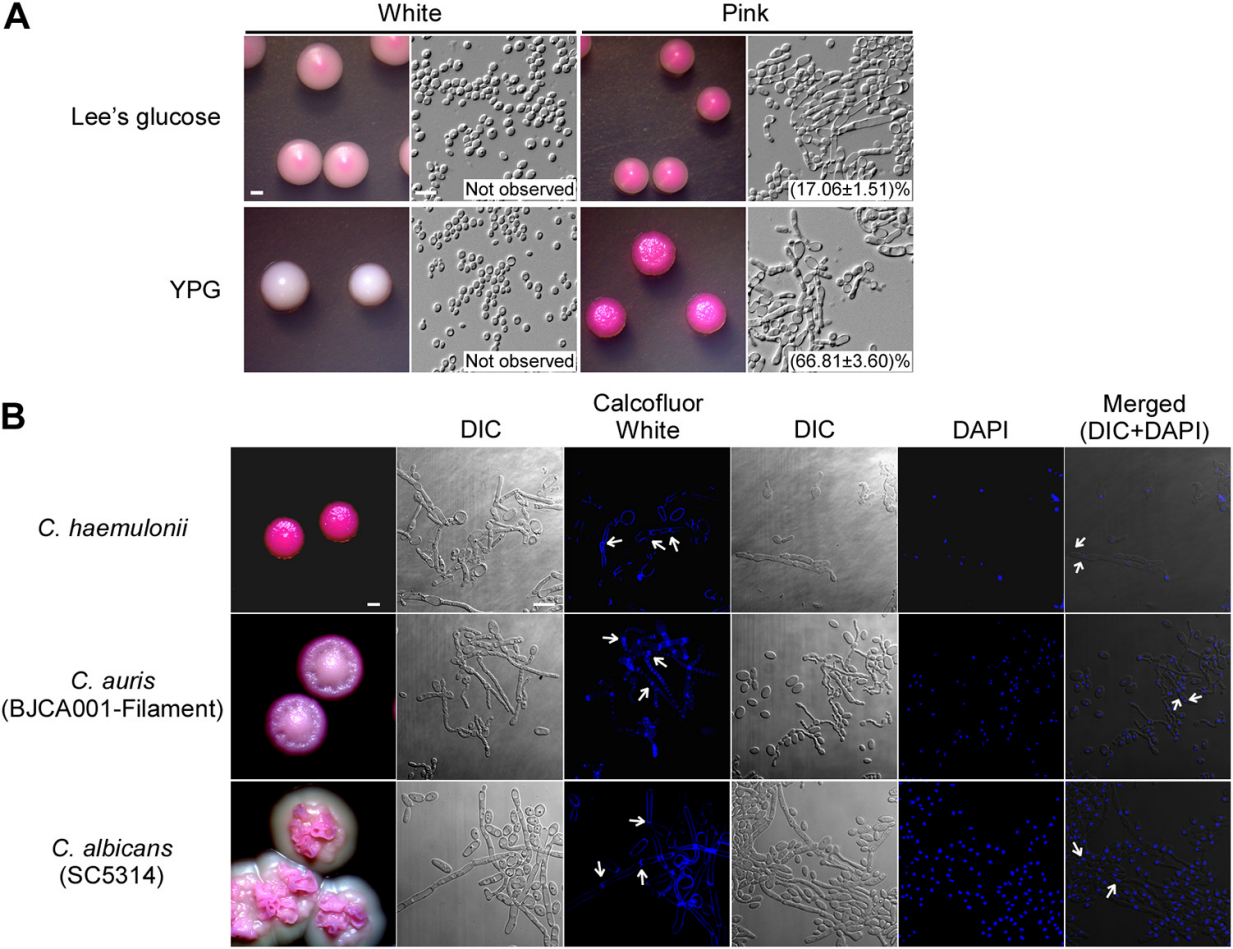
Morphologies of white and pink phenotypes observed on Lee’s glucose and YPG agar containing phloxine B. (A) Strain CH001 was used. White and pink cells were plated on Lee’s glucose or YPG media for 5 days of growth at 25°C. On Lee’s glucose agar, phloxine B dyestained white cells light pink and pink cells dark pink; on YPG agar, the white colonies remained white and the pink colonies were stained dark pink. Filamentous growth of the pink phenotype was promoted under both culture conditions. No filament cells were observed in white colonies. Percentages of filament cells for each sample are shown in the figure. Scale bar for colonies, 1 mm; scale bar for cells, 10 μm. (B) Calcofluor White and DAPI staining of *C. haemulonii* CH001, C. auris BJCA001 and C. albicans SC5314. *C. haemulonii* cells were grown in liquid YPG medium at 25°C for 48 h. *C auris* cells were grown on YPD medium at 25°C for 5 days. C. albicans cells were grown in liquid YPD plus 10% FBS medium at 37°C for 24 h. White arrows indicate septin rings or nuclei. Scale bar for colonies, 1 mm; scale bar for cells, 10 μm.

Yeast-filament switching is the most common strategy by which *Candida* species rapidly adapt to diverse environments and increase their virulence. C. auris, a close relative of C. haemulonii in the Metschnikowiaceae clade, has recently been reported to undergo filamentous growth on YPD medium at 25°C (27). However, under these culture conditions, no filamentous phenotype has been observed in C. haemulonii. Since the yeast-filament switch of *Candida* species is often associated with metabolism alterations, we investigated the effects of various carbon sources on the development of filaments in C. haemulonii. The results showed that C. haemulonii cells of the pink phenotype exclusively formed highly wrinkled colonies containing elongated filaments when they were grown on a yeast-peptone-glycerol (YPG) medium plate at 25°C. A relatively low proportion of filaments were also observed in pink cells grown on Lee’s glucose plates (Fig. 3A). However, no filamentous cells were observed in cells of the white phenotype under all tested culture conditions.

In C. auris, temperature fluctuation from high to low has been demonstrated to promote yeast-filament switching (27). We also found that the fluctuation of temperature from 37°C to 25°C could promote filamentous growth of the white phenotype in C. haemulonii. However, this white-derived filamentous phenotype was unstable and quickly reverted to the yeast form after two additional days of incubation (data not shown). These results indicated that both the white and pink phenotypes of C. haemulonii can undergo filamentous growth; however, only the filamentous phenotype formed by pink cells (hereafter referred to as “filament cells”) was stable. Taken together, the three cell types (white, pink, and filament cells) form a novel switching system that combines white-pink switching and yeast-filament switching.

Differential interference contrast microscopy and Calcofluor White staining demonstrated that C. haemulonii filaments elongated from round or ellipsoidal mother cells and had chitin-containing septa at the junctions between compartments. DAPI staining indicated that the filaments are often multicellular. Filament cells of C. haemulonii were morphologically more similar to the filaments of C. auris than those of C. albicans. The diameters of filaments produced by C. haemulonii, C. auris, and C. albicans were (1.4–2.4 μm), (1.5–2.3 μm), and (2.1–3.2 μm), respectively. The percentages of filament cells containing branches were 9, 23, and 48%, respectively. Therefore, filaments produced by C. haemulonii or C. auris had a thinner diameter and were shorter and less branched than those produced by C. albicans (Fig. 3B).

### Effects of temperature on maintenance of the filamentous phenotype.

The pink phenotype exhibited severe growth defects at physiological temperature (37°C). We further found that filament cells displayed a growth defect similar to that of the pink phenotype at 37°C (Fig. S3 in the supplemental material). To test the effects of temperature on the maintenance of the filamentous phenotype, filament cells derived from the wrinkled colony were plated on YPG agar and incubated at 25 or 30°C. After 8 days of incubation, most of the colonies incubated at 25°C were observed to be wrinkled and 68.88 ± 1.80% of the cells maintained the filamentous phenotype. However, at 30°C, more than 80% of the filament cells converted to yeast form cells, which generated “blebs” on the surfaces of the wrinkled colonies (Fig. S4A). Similar phenomena were observed when filament cells were incubated in liquid YPG medium. After 48 h of incubation, 26.86 ± 1.07% of the cells maintained a filamentous phenotype at 25°C, whereas most of the filament cells (>90%) switched to yeast form cells at 30°C (Fig. S4B). These results showed that the filamentous phenotype was more stable at 25°C than at 30°C. These results suggested that similar to the inhibitory effect of high temperatures on filamentation in C. auris, elevated temperatures also promote filament-yeast switching in C. haemulonii.

### Global gene expression profiles of *C. haemulonii* white and pink cells.

**FIG 4:**
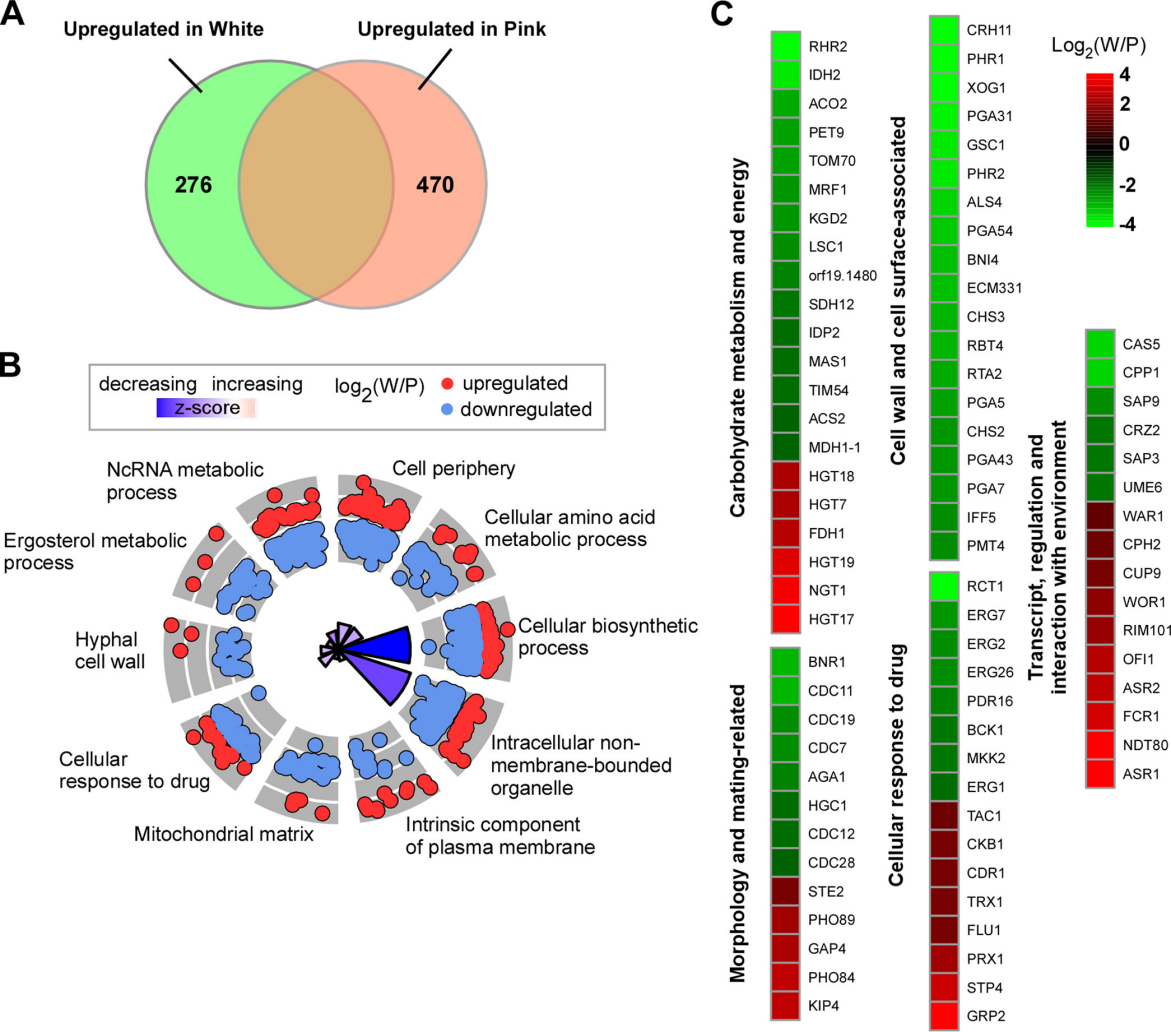
Differential gene expression (DGE) profiles of *C. haemulonii* in white and pink cells. Analysis of DGE was performed using HiSat2 and the Stringtie pipeline. (A). Venn diagram depicting differentially expressed genes. A 1.5-fold difference cutoff and false discovery rates (FDRs) < 0.05 were used to define differentially expressed genes. (B) GO enrichment analysis of differentially expressed genes was conducted. The GO enrichment analysis was performed with Gene Ontology Consortium (http://www.geneontology.org/). The pheatmap and GOplot packages for R were used to visualize clustering. Red or blue circles represent upregulated or downregulated genes in white or filament cells, respectively. The inner cycle bars represent statistical significance. (C) R package heatmap was used to depict selected differentially expressed genes. Functional categories of genes are indicated. Log_2_(W/P), Log_2_ (read counts of white cells/read counts of pink cells).

To further reveal the differences between the white and pink cells, RNA sequencing (RNA-seq) analysis was performed. The filament phenotype was not investigated because filament cells were not stable under the culture conditions used. Using a 1.5-fold cutoff, a total of 746 genes were differentially expressed in white and pink cells (Fig. 4A). Among these identified genes, 470 were upregulated in pink cells and 276 were upregulated in white cells. A more detailed analysis of the differentially expressed genes is shown in Fig. 4B and C and Data set S1 in the supplemental material. As expected, a subset of genes associated with carbohydrate metabolism and energy in C. albicans were differentially expressed between C. haemulonii white and pink cells. Genes belonging to the Krebs cycle pathway (*IDH2*, *ACO2*, *KGD2*, *IDP2*, and *MDH1-1*) were upregulated in pink cells, whereas a range of glucose transporter encoding genes (*HGT7*, *HGT17*, *HGT18*, and *HGT19*) were upregulated in white cells (30, 31). Several morphology-related genes were differentially expressed between the white and pink cell types. For example, *CDC11* (encoding a septin), *HGC1* (encoding a hyphal-specific G1 cyclin-related protein), *AGA1* (encoding an agglutinin) and secreted aspartyl proteinase encoding genes (*SAP3*, *SAP9*) were expressed at higher levels in pink cells, while *OFI1* (encoding a phenotypic switching regulator), and phosphate transporter encoding genes (*PHO84*, *PHO89*) were strongly enriched in white cells (18, 32–36). Moreover, some transcriptional regulator-encoding genes that are involved in filamentous regulation in C. albicans were differentially expressed. For example, *UME6*, which encodes a positive regulator of filamentation in C. albicans, was upregulated in pink cells, and the negative regulators in C. albicans Cup9 and Fcr1 exhibited higher expression in white cells (37–39).

A large subset of cell wall-associated and GPI-anchored protein-encoding genes were differentially expressed between white and pink cells. *ALS4* (encoding a GPI-anchored adhesin), *CSA1* (encoding a hyphal-specific surface antigen), *RBT4* (encoding a hyphal-specific cell wall protein), *PGA31*, and *PGA54* were upregulated in pink cells (40–43). Many genes involved in cell wall integrity, including chitin synthase encoding genes (*CHS2*, *CHS3*, and *CHS4*), *CRH11* (encoding a GPI-anchored cell wall transglycosylase), *XOG1* (encoding an exo-1,3-β-glucanase), and *PMT4* (encoding a protein mannosyltransferase) showed higher expression in pink cells (44–47). Strikingly, a subset of drug resistance-related genes (e.g., *ERG1*, *ERG2*, *ERG7*, *ERG26*, and *PDR16*, and *RCT1*) were significantly enriched in pink cells (48, 49). In addition, we verified the RNA-Seq data with quantitative RT-PCR to measure the expression of 11 identified differentially expressed genes. As shown in Fig. S5 in the supplemental material, the expression patterns of these genes were generally consistent with those of the RNA-Seq assays, indicating that the RNA-Seq analysis is reliable and valid.

### CuSO_4_ tolerance of *C. haemulonii* white, pink, and filament cells.

**FIG 5:**
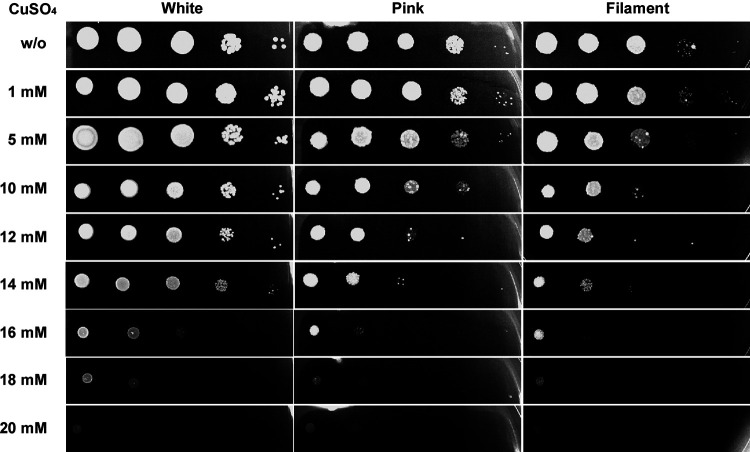
Inhibitory effect of CuSO_4_ on the growth of *C. haemulonii* white, pink, and filament cells at 25°C. The *C. haemulonii* strain was adjusted to 5 × 10^8^ cells/ml, and 10-fold serial dilutions of cells (2 μl) were spotted onto YPD and YPD agar containing serial concentrations of CuSO_4_ for 2 days of growth. w/o: without CuSO_4_.

The transition metal copper (Cu) is a potentially toxic metal, and various forms of Cu have been widely used for biological control (50, 51). Therefore, we investigated the inhibitory effect of CuSO_4_ on the different phenotypes of C. haemulonii. At 25°C, the order of sensitivity to CuSO_4_ from highest to lowest was filament cells > pink cells > white cells (Fig. 5). When increased to 20 mM, CuSO_4_ completely inhibit the growth of all three phenotypes of C. haemulonii. Compared with C. auris, which could not grow in the presence of 5 mM CuSO_4_, C. haemulonii exhibited much higher tolerance to CuSO_4_ (52). In addition, we investigated CuSO_4_ tolerance of the white and opaque cells in C. albicans (Fig. S6). The results indicate that C. albicans is even more resistant to CuSO_4_ than C. haemulonii. Both white cells and opaque cells could grow in the presence of 20 mM CuSO_4_, and white cells exhibited higher copper tolerance than opaque cells.

### White, pink, and filament cells exhibit different SAP activities and virulence.

**FIG 6:**
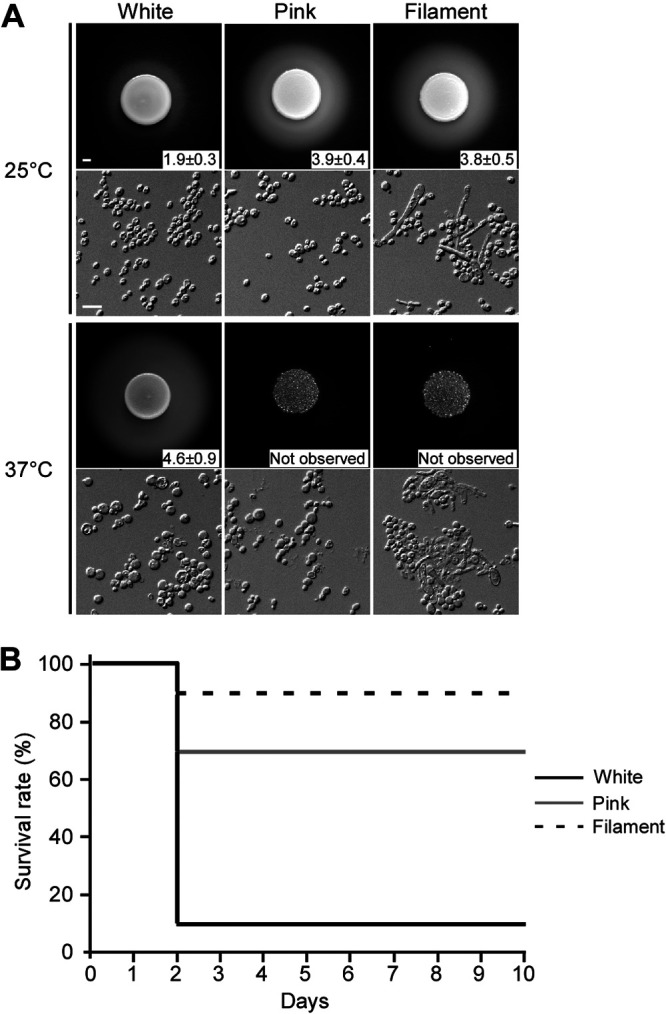
SAP activities of white, pink, and filament cells, and virulence in a G. mellonella infection model. (A) SAP activity. Cells of each cell type (5 × 10^6^ cells) in 5 μl double-distilled water were spotted on YCB-BSA medium and incubated at 25°C or 37°C for 3 days. The width of the white precipitation zones representing SAP activity was measured and indicated. Cellular images of the corresponding spots are shown below. Scale bar for spots, 1 mm; scale bar for cells, 10 μm. (B) Survival rates of G. mellonella infected by white, pink, and filament cells of strain CH001 at 25°C. Cells of each cell type (2 × 10^5^ cells) in 10 μl double-distilled water were injected into larvae of G. mellonella. For each cell type, 10 larvae were used for infection.

SAPs are an important virulence factor that often represents the ability of pathogenic fungi to adhere to or invade host niches (53). We therefore investigated the SAP activity of the three different cell types using the yeast carbon base (YCB)-bovine serum albumin (BSA) assay. As shown in Fig. 6A, both pink and filament cells exhibited higher SAP activity than white cells at 25°C, as indicated by the size of the white halos of precipitated BSA. As expected, C. albicans opaque cells showed higher SAP activity than white cells (Fig. S7A in the supplemental material). However, at 37°C, C. haemulonii white cells exhibited higher SAP activity than at 25°C. Pink and filament cells did not show any SAP activity due to their growth defect at 37°C. These results indicate that both environmental temperature and phenotypic switching modulate SAP activity. Since morphological diversity is often associated with pathogenesis in *Candida* species, we further examined virulence of the different cell types of C. haemulonii and C. albicans by using a Galleria mellonella infection model. As shown in Fig. 6B and S7B, C. albicans opaque cells are more virulent than white cells, and the order of virulence observed in C. haemulonii from highest to lowest was white cells > pink cells > filament cells. Taken together, these results imply that distinct cell types of fungal pathogens often have differential virulence characteristics during host tissue colonization and systemic infection.

## DISCUSSION

Phenotypic plasticity is a striking feature of pathogenic *Candida* species. In this study, we report two phenotypic switching systems in the fungal pathogen C. haemulonii: white-pink switching and pink-filament switching. The two switching systems involve three distinct phenotypes, including white, pink, and filament cells. White and pink cells are two yeast form phenotypes, which differ in terms of the color of colonies formed on phloxine B-containing media, their gene expression profiles, SAP activity, and virulence. The bistable switch between the white and pink cell types is similar to the white-opaque switch in C. albicans (22). Since a subset of C. haemulonii clinical strains were discovered to undergo the white-pink switching, this phenotypic transition may be a general feature of C. haemulonii in nature. An additional pink-filament cell switch system is triggered by the presence of glycerol and modulated by temperature fluctuation. The environmentally dependent pink-filament switch is nonheritable and is similar to the FC-filament transition in C. auris (27).

Pink and filament cells, but not white cells, can be stained pink on phloxine B-containing agar, which suggests differences in the composition or integrity of the cell wall (54). Furthermore, when cells of the three phenotypes were grown on CuSO_4_-containing agar, they exhibited opposite color intensities. White cells formed dark brown colonies on CuSO_4_, while pink and filament cells formed light brown colonies. This is consistent with previously reported findings in C. glabrata (55). The coloration hierarchy on CuSO_4_-containing agar is associated with the amount of CuS produced by CuSO_4_ reduction (MTII), and therefore represents the Cu resistance capabilities of distinct cell types. These results imply that white cells might have higher Cu resistance than pink and filament cells. Consistent with this, the subsequent CuSO_4_ inhibitory assay confirmed that the order of resistance to CuSO_4_ from highest to lowest is white cells > pink cells > filament cells. Compared with C. auris, which could not grow in the presence of 5 mM CuSO_4_, the distinct phenotypes of C. haemulonii could all tolerate a higher concentration of CuSO_4_ (15). Distinct cell wall features and Cu resistance could benefit specific cell types in terms of their survival and persistence within diverse host niches.

Global transcriptomic analysis revealed that a range of Krebs cycle-associated genes that were upregulated in C. albicans opaque cells (e.g., *KGD2*, *IDP2* and *MDH1-1*) exhibited higher expression levels in C. haemulonii pink cells (30). Genes encoding glucose transporter (*HGT7*, *HGT17*, *HGT18* and *HGT19*) that were enriched in C. albicans white cells were upregulated in C. haemulonii white cells (31). Moreover, a range of white-opaque associated genes (*SAP3*, *SAP9*, *OFI1*, *PHO84*, *PHO89*, *AGA1*) in C. albicans were differentially expressed between the white and pink cell types of C. haemulonii (18, 32–36). These results suggest that white-pink switch of C. haemulonii may share similar features with white-opaque transition of C. albicans in terms of metabolism and phenotypic regulation processes.

A subset of filamentation-specific genes, filamentation regulator-encoding genes, and cell wall-associated genes were differentially expressed between the white and pink cells of C. haemulonii. *HGC1*, encoding a G1 cyclin-related protein, *UME6*, and three GPI-anchored protein-encoding genes, *ALS4*, *PGA31* and *PGA54*, were upregulated in pink cells of C. haemulonii (33, 37, 40, 43). This was similar to filament cells of C. albicans and C. auris, indicating that the pink phenotype has the capacity to develop filament cells. In addition, white and pink cells exhibited highly differential expression of cell wall synthesis-related genes (e.g., *CHS2*, *CHS3*, *CHS4*, *PMT4*, *CRH11*, and *XOG1*) (47–47). Cell wall variation might be an important strategy used by C. haemulonii to adhere to the host cell surface, adapt to the host environment, and escape the host immune response. Consistently, SAP activity and G. mellonella infection assays indicated that white, pink, and filament cells have differential virulence characteristics during host tissue colonization and systemic infection.

Increased expression of a number of ergosterol biosynthesis genes (*ERG1*, *ERG2*, *ERG7*, *ERG26*, *PDR16*, and *RCT1*) were also observed in pink cells of C. haemulonii, suggesting that pink cells might have higher levels of multidrug resistance than white cells (48, 49). Further investigation needs to be performed to evaluate the multidrug resistance of the distinct cell types. Although both white-pink switching in C. haemulonii and the white-opaque transition in C. albicans are heritable and contribute to environmental adaptation and infection virulence, there are still distinct features between the two phenotypic switching systems. First, the colony and cellular morphologies of C. albicans opaque cells and C. haemulonii pink cells was distinguishable. C. albicans opaque cells were elongated and formed rougher colonies on Lee’s glucose agar medium, while C. haemulonii pink cells were round and formed smooth colonies. Second, C. albicans white cells can undergo robust filamentous growth under many laboratory conditions, such as elevated CO_2_ levels, neutral pH, physiological temperature (37°C), and *N*-acetylglucosamine exposure (20). Conversely, white cells of C. haemulonii were not observed to form filaments under these conditions, while pink cells undergo filamentous growth when cultured in glycerol-containing medium. Third, although many genes encoding the homologs of the C. albicans mating regulators were upregulated in C. haemulonii pink and filament cells, no mating phenotype or behavior was observed. Taken together, the possible mechanism and physiological significance underlying morphological switching in C. haemulonii need to be investigated further.

The switch between pink and filament cells in C. haemulonii is similar to the transition between the FC yeast and filamentous form in C. auris in terms of their nonheritable property (27), temperature dependence, and filament morphology. However, there are some differences between the two cell types. Low temperatures promote the FC yeast to filamentous form transition in C. auris. In C. haemulonii, the presence of glycerol induces robust filamentous growth. There are two possible explanations for how glycerol drives filament development. One explanation is associated with the well-established role of glycerol in osmotic and oxidative pressure (56, 57). Global gene expression analysis indicated that a subset of oxidative stress response genes (e.g., *TSA1*, *GZF3*) and aerobic metabolism-related genes (e.g., *IDH2*, *ACO2*, *IDP2*, *KGD2*) were enriched in the pink cells, consistent with this explanation. The second hypothesis is that internal glycerol is required for GPI anchor synthesis, which is necessary for the cross-linking of mannoproteins and adhesins to the cell surface. Cell surface deposition has been demonstrated to contribute to the capability of cells for undergoing filamentation (58). This notion might be supported by the identification of significantly enriched GPI-anchored proteins in C. haemulonii pink cells via global gene expression analysis.

Since its original clinical characterization from human blood in 1984, no filamentous phenotype has been discovered in any isolates of C. haemulonii. Sipiczki et al. (2016) found *C. vulturna*, a novel species related to C. haemulonii, could produce invasive bundles of pseudohyphae (intrusions into the medium) beneath the colonies (59). However, no filament cells were observed in samples taken from colonies on the medium plate. In this study, we discovered a typical filamentous “filament cell” phenotype in C. haemulonii colonies grown on YPG medium plates. The filament cells exhibit several characteristics of the pink cell phenotype, including a pink color on phloxine B-containing agar, a very light brown color on CuSO_4_-containing agar, and defective growth at 37°C. Moreover, elevated temperatures promote the morphological transition from the filament cell type to the pink cell type. Taken together, we propose a novel yeast-filament switching system in C. haemulonii. Unlike most *Candida* species, C. haemulonii is better at superficial infections than systemic infections (10–13). Low temperatures induced filamentation and SAP accumulation in C. haemulonii, thereby facilitating its commensal lifestyle on host skin.

## MATERIALS AND METHODS

### Strains and culture conditions.

C. haemulonii, C. auris, and C. albicans strains were routinely cultured in YPD medium (20 g/liter peptone, 10 g/liter yeast extract, 20 g/liter glucose). YPG (20 g/liter peptone, 10 g/liter yeast extract, 20 ml/liter glycerol), YPD + 1 mM CuSO_4_, and modified Lee’s glucose media (25) were used for the morphological assays. Solid media were supplemented with 2% Bacto-Agar and 5 μg/ml phloxine B dye. Yeast extract was purchased from the Angel Company (Hubei, China). Peptone was from Oxoid Ltd. Company (Hants, UK). Glucose and glycerol were purchased from Beijing Chemical Works (Beijing, China), and phloxine B was from Sigma-Aldrich (St. Louis, MO). For plating experiments, cells were diluted into double-distilled water at a concentration of 1,000 CFU/ml. Then, 0.1 ml of the suspension was spread on the different solid media and cultured at 25, 30, or 37°C for 5–9 days. For the CuSO_4_ inhibition assays, YPD and YPD agar containing serial concentrations of CuSO_4_ was used. YCB agar medium containing 0.2% BSA as the sole nitrogen source was used for the SAP activity assays. All strains used in this study are listed in Table S1 in the supplemental material.

### Morphological switching assays.

For the phenotypic switching assays, cells of the white or pink phenotypes were plated onto YPD+phloxine B medium and incubated at 25°C for 6 days. The switching frequency was defined as the number of colonies with an alternative phenotype divided by the number of total colonies. Data were presented as the switching frequency percentage ± standard deviation.

### Phylogenetic analysis.

The ITS sequences of C. haemulonii CH001 and previously reported isolates were aligned using MEGA, Version 6.0. Minimum evolution phylogenetic trees were constructed with the neighbor-joining method, bootstrapped with 1,000 replicates. C. auris strain CBS 10913^T^ was used as an outgroup, whereas C. haemulonii CBS 5149^T^, Candida pseudohaemulonii CBS 10004^T^, Candida duobushaemulonii CBS 7798^T^, Candida vulturna CBS 14366^T^, and Candida haemulonii *var. vulnera* served as comparators. The ITS sequences of the reported strains were acquired from the GenBank (https://www.ncbi.nlm.nih.gov/) or CGD (http://www.candidagenome.org/) databases directly or extracted from the genome sequences.

### DAPI and Calcofluor White staining assays.

Filament cells of *C. haemulonii* were grown in liquid YPG medium at 25°C for 48 h, collected by centrifugation, washed, and resuspended in double-distilled water. For the induction of filaments, C. albicans or C. auris cells were inoculated in liquid YPD + 10% fetal bovine serum (FBS) or YPD agar media for 24 h (37°C) or 5 days (25°C) of growth. Differential interference contrast optics were used for standard cellular morphology assays. The cells were fixed and stained with 4’,6-diamidino-2-phenylindole (DAPI; Sigma-Aldrich, Inc., Beijing, China) to visualize the nucleus, and stained with Calcofluor White (Sigma-Aldrich, Inc.) to visualize septa/chitin. The images were taken by Zeiss-LSM880 with Airy scan (original magnification, ×630).

### RNA-seq analysis and quantitative real-time PCR.

RNA-seq analysis was performed as described previously (60). Briefly, a single colony of white or pink cells was inoculated in liquid YPD medium for 36 h of growth with shaking at 25°C. Total RNA was extracted using GeneJET RNA Purification kits according to the manufacturer’s instructions. RNA-seq was conducted by Berry Genomics Co., (Beijing, China) on the Illumina NovaSeq platform. Approximately 6 million (M) reads were obtained by sequencing each library. The clean reads were aligned to the reference sequence (*C. haemulonii* genomic sequence data from the NCBI database, GCA_002926055.1) via the software HiSat2 v2.0.5 with default parameters (61). Differentially expressed genes were analyzed with the DESeq2 R package (62). Three biological repeats were performed. The RNA-seq data set has been deposited into the NCBI Gene Expression Omnibus (GEO) portal. The GO enrichment analysis was performed with Gene Ontology Consortium (http://www.geneontology.org/). The heatmap and GOplot packages for R were used to visualize clustering (63).

For the quantitative real-time PCR (qRT-PCR) analysis, 1 μg of total RNA per sample was used to synthesize cDNA with RevertAid Reverse Transcriptase (Thermo Scientific, Inc.) following the manufacturer’s recommendations. The primers used for qRT-PCR are listed in Table S2 in the supplemental material. Quantification of the transcripts was performed in a Bio-Rad CFX96 real-time PCR detection system using SYBR green. C. haemulonii ACT1 served as a housekeeping gene. The expression levels of each experimental sample were normalized to that of *ACT1*.

### Galleria mellonella infection assays.

G. mellonella in the final instar larval stage were purchased from Tianjin Huiyu Biological Technology Co. (Tianjin, China). Larvae of a similar size (0.3–0.4 g) were used for infection assays. *C. haemulonii* white and pink cells, or C. albicans white and opaque cells were cultured on YPD medium at 25°C for 5 days. *C. haemulonii* filament cells were grown on YPG medium at 25°C for 5 days. Cells of each phenotype (5 × 10^5^ for *C. haemulonii* or 1 × 10^6^ for C. albicans) in 10 μl 1 × PBS were injected into each larva using a syringe as described previously (52). After injection, the larvae were placed in plastic culture dishes and incubated at 25°C in the dark.

### Accession Number.

The RNA-seq data set has been deposited into the NCBI Gene Expression Omnibus (GEO) portal (accession number GSE185568).

## References

[1] Chowdhary A, Sharma C, Meis JF. 2017. *Candida auris*: a rapidly emerging cause of hospital-acquired multidrug-resistant fungal infections globally. PLoS Pathog 13:e1006290. doi:10.1371/journal.ppat.1006290. 28542486 PMC5436850

[2] Du H, Bing J, Hu T, Ennis CL, Nobile CJ, Huang G. 2020. *Candida auris*: Epidemiology, biology, antifungal resistance, and virulence. PLoS Pathog 16:e1008921. doi:10.1371/journal.ppat.1008921. 33091071 PMC7581363

[3] Jeffery-Smith A, Taori SK, Schelenz S, Jeffery K, Johnson EM, Borman A, Manuel R, Colin SB. 2018. *Candida auris*: a review of the literature. Clin Microbiol Rev 31:e00029-17. doi:10.1128/CMR.00029-17. 29142078 PMC5740969

[4] Saris K, Meis JF, Voss A. 2018. Candida auris. Curr Opin Infect Dis 31:334–340. doi:10.1097/QCO.0000000000000469. 29878905

[5] Lavarde V, Daniel F, Saez H, Arnold M, Faguer B. 1984. Peritonite mycosique a *Torulopsis haemulonii*. Bull Soc Fr Mycol Med 13:173–176.

[6] Chowdhary A, Sharma C, Duggal S, Agarwal K, Prakash A, Singh PK, Jain S, Kathuria S, Randhawa HS, Hagen F, Meis JF. 2013. New clonal strain of *Candida auris*, Delhi, India. Emerg Infect Dis 19:1670–1673. doi:10.3201/eid1910.130393. 24048006 PMC3810747

[7] Lee WG, Shin JH, Uh Y, Kang MG, Kim SH, Park KH, Jang HC. 2011. First three reported cases of nosocomial fungemia caused by *Candida auris*. J Clin Microbiol 49:3139–3142. doi:10.1128/JCM.00319-11. 21715586 PMC3165631

[8] Magobo RE, Corcoran C, Seetharam S, Govender NP. 2014. *Candida auris*-associated candidemia, South Africa. Emerg Infect Dis 20:1250–1251. doi:10.3201/eid2007.131765. 24963796 PMC4073876

[9] Chowdhary A, Kumar VA, Sharma C, Prakash A, Agarwal K, Babu R, Dinesh KR, Karim S, Singh SK, Hagen F, Meis JF. 2014. Multidrug-resistant endemic clonal strain of *Candida auris* in India. Eur J Clin Microbiol Infect Dis 33:919–926. doi:10.1007/s10096-013-2027-1. 24357342

[10] Kim S, Ko KS, Moon SY, Lee MS, Lee MY, Son JS. 2011. Catheter-related candidemia caused by *Candida haemulonii* in a patient in long-term hospital care. J Korean Med Sci 26:297–300. doi:10.3346/jkms.2011.26.2.297. 21286025 PMC3031018

[11] Rodero L, Cuenca-Estrella M, Córdoba S, Cahn P, Davel G, Kaufman S, Guelfand L, Rodríguez-Tudela JL. 2002. Transient fungemia caused by an amphotericin B-resistant isolate of *Candida haemulonii*. J Clin Microbiol 40:2266–2269. doi:10.1128/JCM.40.6.2266-2269.2002. 12037106 PMC130759

[12] Ruan SY, Kuo YW, Huang CT, Hsiue HC, Hsueh PR. 2010. Infections due to *Candida haemulonii*: species identification, antifungal susceptibility and outcomes. Int J Antimicrob Ag 35:85–88. doi:10.1016/j.ijantimicag.2009.08.009. 19786341

[13] Crouzet J, Sotto A, Picard E, Lachaud L, Bourgeois N. 2011. A case of *Candida haemulonii* osteitis: clinical features, biochemical characteristics, and antifungal resistance profile. Clin Microbiol Infect 17:1068–1070. doi:10.1111/j.1469-0691.2011.03471.x. 21375662

[14] Ramos LS, Figueiredo-Carvalho MH, Barbedo LS, Ziccardi M, Chaves AL, Zancope-Oliveira RM, Pinto MR, Sgarbi DB, Dornelas-Ribeiro M, Branquinha MH, Santos AL. 2015. *Candida haemulonii* complex: species identification and antifungal susceptibility profiles of clinical isolates from Brazil. J Antimicrob Chemother 70:111–115. doi:10.1093/jac/dku321. 25134720

[15] Hou X, Xiao M, Chen SC, Wang H, Cheng JW, Chen XX, Xu ZP, Fan X, Kong F, Xu YC. 2016. Identification and antifungal susceptibility profiles of *Candida haemulonii* species complex clinical isolates from a multicenter study in China. J Clin Microbiol 54:2676–2680. doi:10.1128/JCM.01492-16. 27535688 PMC5078542

[16] Khan ZU, Al-Sweih NA, Ahmad S, Al-Kazemi N, Khan S, Joseph L, Chandy R. 2007. Outbreak of fungemia among neonates caused by *Candida haemulonii* resistant to amphotericin B, itraconazole, and fluconazole. J Clin Microbiol 45:2025–2027. doi:10.1128/JCM.00222-07. 17428940 PMC1933024

[17] Muro MD, Motta FDA, Burger M, Melo ASDA, Dalla-Costa LM. 2012. Echinocandin resistance in two *Candida haemulonii* isolates from pediatric patients. J Clin Microbiol 50:3783–3785. doi:10.1128/JCM.01136-12. 22895037 PMC3486200

[18] Lan C-Y, Newport G, Murillo LA, Jones T, Scherer S, Davis RW, Agabian N. 2002. Metabolic specialization associated with phenotypic switching in *Candida albicans*. Proc Natl Acad Sci USA 99:14907–14912. doi:10.1073/pnas.232566499. 12397174 PMC137518

[19] Noble SM, Gianetti BA, Witchley JN. 2017. *Candida albicans* cell-type switching and functional plasticity in the mammalian host. Nat Rev Microbiol 15:96–108. doi:10.1038/nrmicro.2016.157. 27867199 PMC5957277

[20] Huang G. 2012. Regulation of phenotypic transitions in the fungal pathogen *Candida albicans*. Virulence 3:251–261. doi:10.4161/viru.20010. 22546903 PMC3442837

[21] Biswas S, Van Dijck P, Datta A. 2007. Environmental sensing and signal transduction pathways regulating morphopathogenic determinants of *Candida albicans*. Microbiol Mol Biol Rev 71:348–376. doi:10.1128/MMBR.00009-06. 17554048 PMC1899878

[22] Slutsky B, Staebell M, Anderson J, Risen L, Pfaller M, Soll DR. 1987. White-opaque transition: a second high-frequency switching system in *Candida albicans*. J Bacteriol 169:189–197. doi:10.1128/jb.169.1.189-197.1987. 3539914 PMC211752

[23] Lohse MB, Johnson AD. 2009. White-opaque switching in *Candida albicans*. Curr Opin Microbiol 12:650–654. doi:10.1016/j.mib.2009.09.010. 19853498 PMC2812476

[24] Soll DR. 2009. Why does *Candida albicans* switch? FEMS Yeast Res 9:973–989. doi:10.1111/j.1567-1364.2009.00562.x. 19744246

[25] Xie J, Tao L, Nobile CJ, Tong Y, Guan G, Sun Y, Cao C, Hernday AD, Johnson AD, Zhang L, Bai F-Y, Huang G. 2013. White-opaque switching in natural *MTL*a/alpha isolates of *Candida albicans*: evolutionary implications for roles in host adaptation, pathogenesis, and sex. PLoS Biol 11:e1001525. doi:10.1371/journal.pbio.1001525. 23555196 PMC3608550

[26] Guan G, Xie J, Tao L, Nobile CJ, Sun Y, Cao C, Tong Y, Huang G. 2013. Bcr1 plays a central role in the regulation of opaque cell filamentation in *Candida albicans*. Mol Microbiol 89:732–750. doi:10.1111/mmi.12310. 23808664 PMC3758918

[27] Yue H, Bing J, Zheng Q, Zhang Y, Hu T, Du H, Wang H, Huang G. 2018. Filamentation in *Candida auris*, an emerging fungal pathogen of humans: passage through the mammalian body induces a heritable phenotypic switch. Emerg Microbes Infect 7:188. doi:10.1038/s41426-018-0187-x. 30482894 PMC6258701

[28] Whiteway M, Bachewich C. 2007. Morphogenesis in *Candida albicans*. Annu Rev Microbiol 61:529–553. doi:10.1146/annurev.micro.61.080706.093341. 17506678 PMC4452225

[29] Tao L, Du H, Guan G, Dai Y, Nobile CJ, Liang W, Cao C, Zhang Q, Zhong J, Huang G. 2014. Discovery of a “white-gray-opaque” tristable phenotypic switching system in *Candida albicans*: roles of non-genetic diversity in host adaptation. PLoS Biol 12:e1001830. doi:10.1371/journal.pbio.1001830. 24691005 PMC3972085

[30] Kusch H, Engelmann S, Bode R, Albrecht D, Morschhäuser J, Hecker M. 2008. A proteomic view of *Candida albicans* yeast cell metabolism in exponential and stationary growth phases. Int J Med Microbiol 298:291–318. doi:10.1016/j.ijmm.2007.03.020. 17588813

[31] Fan J, Chaturvedi V, Shen S. 2002. Identification and phylogenetic analysis of a glucose transporter gene family from the human pathogenic yeast *Candida albicans*. J Mol Evol 55:336–346. doi:10.1007/s00239-002-2330-4. 12187386

[32] Warenda AJ, Konopka JB. 2002. Septin function in *Candida albicans* morphogenesis. Mol Biol Cell 13:2732–2746. doi:10.1091/mbc.e02-01-0013. 12181342 PMC117938

[33] Zheng X, Wang Y, Wang Y. 2004. Hgc1, a novel hypha-specific G1 cyclin-related protein regulates *Candida albicans* hyphal morphogenesis. EMBO J 23:1845–1856. doi:10.1038/sj.emboj.7600195. 15071502 PMC394249

[34] White TC, Miyasaki SH, Agabian N. 1993. Three distinct secreted aspartyl proteinases in *Candida albicans*. J Bacteriol 175:6126–6133. doi:10.1128/jb.175.19.6126-6133.1993. 8407785 PMC206706

[35] Monod M, Hube B, Hess D, Sanglard D. 1998. Differential regulation of *SAP8* and *SAP9*, which encode two new members of the secreted aspartic proteinase family in *Candida albicans*. Microbiology 144:2731–2737. doi:10.1099/00221287-144-10-2731. 9802014

[36] Du H, Li X, Huang G, Kang Y, Zhu L. 2015. The zinc-finger transcription factor, Ofi1, regulates white-opaque switching and filamentation in the yeast *Candida albicans*. Acta Biochim Biophys Sin (Shanghai) 47:335–341. doi:10.1093/abbs/gmv011. 25757952

[37] Banerjee M, Thompson DS, Lazzell A, Carlisle PL, Pierce C, Monteagudo C, López-Ribot JL, Kadosh D. 2008. UME6, a novel filament-specific regulator of *Candida albicans* hyphal extension and virulence. Mol Biol Cell 19:1354–1365. doi:10.1091/mbc.e07-11-1110. 18216277 PMC2291399

[38] Nantel A, Dignard D, Bachewich C, Harcus D, Marcil A, Bouin A-P, Sensen CW, Hogues H, van Het Hoog M, Gordon P, Rigby T, Benoit F, Tessier DC, Thomas DY, Whiteway M. 2002. Transcription profiling of *Candida albicans* cells undergoing the yeast-to-hyphal transition. Mol Biol Cell 13:3452–3465. doi:10.1091/mbc.e02-05-0272. 12388749 PMC129958

[39] Talibi D, Raymond M. 1999. Isolation of a putative *Candida albicans* transcriptional regulator involved in pleiotropic drug resistance by functional complementation of a pdr1 pdr3 mutation in *Saccharomyces cerevisiae*. J Bacteriol 181:231–240. doi:10.1128/JB.181.1.231-240.1999. 9864335 PMC103554

[40] Hoyer LL, Payne TL, Hecht JE. 1998. Identification of *Candida albicans* ALS2 and ALS4 and localization of als proteins to the fungal cell surface. J Bacteriol 180:5334–5343. doi:10.1128/JB.180.20.5334-5343.1998. 9765564 PMC107581

[41] Lamarre C, Deslauriers N, Bourbonnais Y. 2000. Expression cloning of the *Candida albicans CSA1* gene encoding a mycelial surface antigen by sorting of *Saccharomyces cerevisiae* transformants with monoclonal antibody-coated magnetic beads. Mol Microbiol 35:444–453. doi:10.1046/j.1365-2958.2000.01715.x. 10652105

[42] Braun BR, Head WS, Wang MX, Johnson AD. 2000. Identification and characterization of TUP1-regulated genes in *Candida albicans*. Genetics 156:31–44. doi:10.1093/genetics/156.1.31. 10978273 PMC1461230

[43] Richard ML, Plaine A. 2007. Comprehensive analysis of glycosylphosphatidylinositol-anchored proteins in *Candida albicans*. Eukaryot Cell 6:119–133. doi:10.1128/EC.00297-06. 17189485 PMC1797948

[44] Mio T, Yabe T, Sudoh M, Satoh Y, Nakajima T, Arisawa M, Yamada-Okabe H. 1996. Role of three chitin synthase genes in the growth of *Candida albicans*. J Bacteriol 178:2416–2419. doi:10.1128/jb.178.8.2416-2419.1996. 8636047 PMC177954

[45] de Groot PW, de Boer AD, Cunningham J, Dekker HL, de Jong L, Hellingwerf KJ, de Koster C, Klis FM. 2004. Proteomic analysis of *Candida albicans* cell walls reveals covalently bound carbohydrate-active enzymes and adhesins. Eukaryot Cell 3:955–965. doi:10.1128/EC.3.4.955-965.2004. 15302828 PMC500891

[46] González MDM, Díez-Orejas R, Molero G, Álvarez AM, Pla J, Pla J, Nombela C, Sánchez-PéArez M. 1997. Phenotypic characterization of a *Candida albicans* strain deficient in its major exoglucanase. Microbiology 143:3023–3032. doi:10.1099/00221287-143-9-3023. 9308184

[47] Kruppa M, Jabra-Rizk MA, Meiller TF, Calderone R. 2004. The histidine kinases of *Candida albicans*: regulation of cell wall mannan biosynthesis. FEMS Yeast Res 4:409–416. doi:10.1016/S1567-1356(03)00201-0. 14734021

[48] Liu TT, Lee REB, Barker KS, Lee RE, Wei L, Homayouni R, Rogers PD. 2005. Genome-wide expression profiling of the response to azole, polyene, echinocandin, and pyrimidine antifungal agents in *Candida albicans*. Antimicrob Agents Chemother 49:2226–2236. doi:10.1128/AAC.49.6.2226-2236.2005. 15917516 PMC1140538

[49] De Deken X, Raymond M. 2004. Constitutive activation of the *PDR16* promoter in a *Candida albicans* azole-resistant clinical isolate overexpressing *CDR1* and *CDR2*. Antimicrob Agents Chemother 48:2700–2703. doi:10.1128/AAC.48.7.2700-2703.2004. 15215129 PMC434214

[50] Festa RA, Helsel ME, Franz KJ, Thiele DJ. 2014. Exploiting innate immune cell activation of a copper-dependent antimicrobial agent during infection. Chem Biol 21:977–987. doi:10.1016/j.chembiol.2014.06.009. 25088681 PMC4170187

[51] Li CX, Gleason JE, Zhang SX, Bruno VM, Cormack BP, Culotta VC. 2015. *Candida albicans* adapts to host copper during infection by swapping metal cofactors for superoxide dismutase. Proc Natl Acad Sci USA 112:E5336–E5342. doi:10.1073/pnas.1513447112. 26351691 PMC4586888

[52] Wang X, Bing J, Zheng Q, Zhang F, Liu J, Yue H, Tao L, Du H, Wang Y, Wang H, Huang G. 2018. The first isolate of *Candida auris* in China: clinical and biological aspects. Emerg Microbes Infec 7:1–9. doi:10.1038/s41426-018-0095-0. 29777096 PMC5959928

[53] Hube B. 1996. Candida albicans secreted aspartyl proteinases. Curr Top Med Mycol 7:55–69. 9504059

[54] Du H, Zheng Q, Bing J, Bennett RJ, Huang G. 2018. A coupled process of same- and opposite-sex mating generates polyploidy and genetic diversity in *Candida tropicalis*. PLoS Genet 14:e1007377. doi:10.1371/journal.pgen.1007377. 29734333 PMC5957450

[55] Lachke SA, Joly S, Daniels K, Soll DR. 2002. Phenotypic switching and filamentation in *Candida glabrata*. Microbiology 148:2661–2674. doi:10.1099/00221287-148-9-2661. 12213913

[56] San José C, Monge RA, Pérez-Díaz R, Pla J, Nombela C. 1996. The mitogen-activated protein kinase homolog *HOG1* gene controls glycerol accumulation in the pathogenic fungus *Candida albicans*. J Bacteriol 178:5850–5852. doi:10.1128/jb.178.19.5850-5852.1996. 8824643 PMC178437

[57] Alonso-Monge R, Navarro-García F, Román E, Negredo AI, Eisman B, Nombela C, Pla J. 2003. The Hog1 mitogen-activated protein kinase is essential in the oxidative stress response and chlamydospore formation in *Candida albicans*. Eukaryot Cell 2:351–361. doi:10.1128/EC.2.2.351-361.2003. 12684384 PMC154845

[58] Verstrepen KJ, Klis FM. 2006. Flocculation, adhesion and biofilm formation in yeasts. Mol Microbiol 60:5–15. doi:10.1111/j.1365-2958.2006.05072.x. 16556216

[59] Sipiczki M, Tap RM. 2016. *Candida vulturna* pro tempore sp. nov., a dimorphic yeast species related to the *Candida haemulonis* species complex isolated from flowers and clinical sample. Int J Syst Evol Microbiol 66:4009–4015. doi:10.1099/ijsem.0.001302. 27411802

[60] Tao L, Zhang Y, Fan S, Nobile CJ, Guan G, Huang G. 2017. Integration of the tricarboxylic acid (TCA) cycle with cAMP signaling and Sfl2 pathways in the regulation of CO_2_ sensing and hyphal development in *Candida albicans*. PLoS Genet 13:e1006949. doi:10.1371/journal.pgen.1006949. 28787458 PMC5567665

[61] Pertea M, Kim D, Pertea GM, Leek JT, Salzberg SL. 2016. Transcript-level expression analysis of RNA-seq experiments with HISAT, StringTie and Ballgown. Nat Protoc 11:1650–1667. doi:10.1038/nprot.2016.095. 27560171 PMC5032908

[62] Love MI, Huber W, Anders S. 2014. Moderated estimation of fold change and dispersion for RNA-seq data with DESeq2. Genome Biol 15:550. doi:10.1186/s13059-014-0550-8. 25516281 PMC4302049

[63] Walter W, Sanchez-Cabo F, Ricote M. 2015. GOplot: an R package for visually combining expression data with functional analysis. Bioinformatics 31:2912–2914. doi:10.1093/bioinformatics/btv300. 25964631

