# Supplementary material for: Phenotypic Switching and Filamentation in Candida haemulonii, an Emerging Opportunistic Pathogen of Humans
Source: Microbiol Spectr. 2021 Dec 8;9(3):e00779-21. doi: 10.1128/Spectrum.00779-21 (PMC8653834; doi:10.1128/Spectrum.00779-21)
Supplement: SUPPLEMENTAL FILE 2 — Supplemental material. Download SPECTRUM00779-21_Supp_1_seq11.pdf, PDF file, 0.7 MB [file spectrum00779-21_supp_1_seq11.pdf]

Supporting Information

Table S1 Strains used in this study.

Strain name	Description	Reference
Candida haemulonii		
CH001	Wild type, a clinical strain of *C. haemulonii* isolated from blood culture	This study
12XY117	Wild type, a clinical strain of *C. haemulonii* isolated from pus culture	[1]
11TJ209	Wild type, a clinical strain of *C. haemulonii* isolated from venous catheter	[1]
13XN013	Wild type, a clinical strain of *C. haemulonii* isolated from venous catheter	[1]
14XN091	Wild type, a clinical strain of *C. haemulonii* isolated from venous catheter	[1]
14NJ254	Wild type, a clinical strain of *C. haemulonii* isolated from blood culture	[1]
13NJ153	Wild type, a clinical strain of *C. haemulonii* isolated from blood culture	[1]
Candida albicans		
SC5314	Wild type strain of *C. albicans*	[2]
BJ1097	Wild type strain of *C. albicans*	[3]
Candida auris		
BJCA001	Wild type, a clinical strain of *C. auris* isolated from the bronchoalveolar lavage fluid	[4]

Reference:

1. Hou, X., Xiao, M., Chen, SC-A., Wang, H., Cheng, J-W., Chen, X-X., Xu, Z-P., Fan, X., Kong, F., Xu, Y-C. (2016) Identification and antifungal susceptibility profiles of *Candida haemulonii* species complex clinical isolates from a multicenter study in China. J Clin Microbiol 54: 2676-2680.

2. Fonzi, W.A., Irwin, M.Y. (1993) Isogenic strain construction and gene mapping in *Candida albicans*. Genetics 134: 717-728.

3. Tao, L., Du, H., Guan, G., Dai, Y., Nobile, C.J., Liang, W., Cao, C., Zhang, Q., Zhong, J., Huang, G. (2014) Discovery of a "white-gray-opaque" tristable phenotypic switching system in *Candida albicans*: roles of non-genetic diversity in host adaptation. PLoS Biol 12: e1001830.

4. Wang, X., Bing, J., Zheng, Q., Zhang, F., Liu, J., Yue, H., Tao, L., Du, H., Wang, Y., Wang, H., Huang, G. (2018) The first isolate of *Candida auris* in China: clinical and biological aspects. Emerg Microbes Infec 7: 1-9.

Table S2. Primers used in this study.

Name	Sequence (5' to 3')	Description
RT-CRH11 fwd	ACCCACTATCGTCTTCCTGT	qRT-PCR
RT-CRH11 rev	TTAGATCTGATGCCTGGGTT	
RT-PHR1 fwd	GAGTATACGCTGTGGACCCT	
RT-PHR1 rev	CCCAACACGTTCGAGTAGTT	
RT-PGA31 fwd	AGACCTTCAACCTCCAAATT	
RT-PGA31 rev	GCCCACAGCAAAGTAGTT	
RT-KIP4 fwd	GTCATAACCTCTTTTCCCAGAT	
RT-KIP4 rev	CGAGAGACCCTTAACGTAGACT	
RT-PHO84 fwd	GCCTGATTCTACTACCACCCT	
RT-PHO84 rev	GGAGAAGAGCCAATAACACACT	
RT-CDC11 fwd	CCTGCAAGACCCTTTCCT	
RT-CDC11 rev	CCTCGTCATATTGGTGCCT	
RT-GRP2 fwd	GGATATCGCCAAGGAAGGT	
RT-GRP2 rev	ACCCTCTTGATATTTGTGCCAT	
RT-STP4 fwd	CTCTCGCTTGATCTCCCTAT	
RT-STP4 rev	TGAACGTGATCATAGCCCT	
RT-RCT1 fwd	CGACAACAACTACCAGTCTTCT	
RT-RCT1 rev	CCAATCTTACCCAAAGTAGAGT	
RT-RIM101 fwd	CTCCACAAACTTCCTTCACAT	
RT-RIM101 rev	GAGTGAGGTTGAGGTTGCT	
RT-CUP9 fwd	GCCTGTCTACCTGTACCAGT	
RT-CUP9 rev	CAACGAGCCTTGTGGGTT	

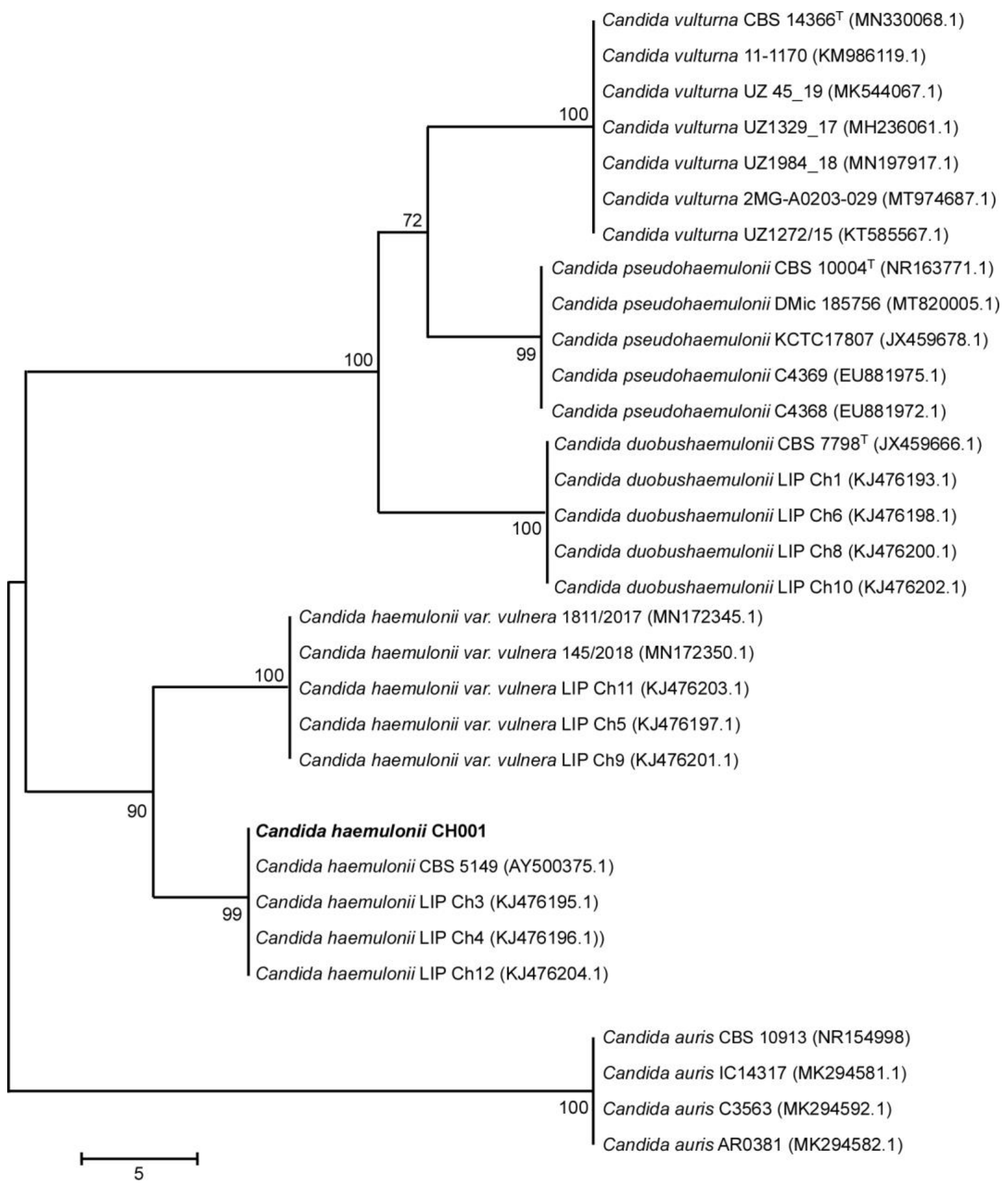

FIG S1 Phylogenetic trees generated through the neighbor-joining method. ITS sequences of nuclear rDNA of *Candida haemulonii* CH001, *C. haemulonii* closely related species, and *Candida auris* were used. The GenBank accession numbers are shown in the brackets. *Candida* species used: *C. haemulonis*, *C. duobushaemulonii*, *C. pseudohaemulonii*, *C. vulturna*, and *C. haemulonii var. vulnera*. The percentages of replicate trees in which the associated taxa clustered

together in the bootstrap test (1,000 replicates) are indicated at the branches. The scale bar indicates the number of inferred substitutions per nucleotide site. Strain CH001 is highlighted in bold.

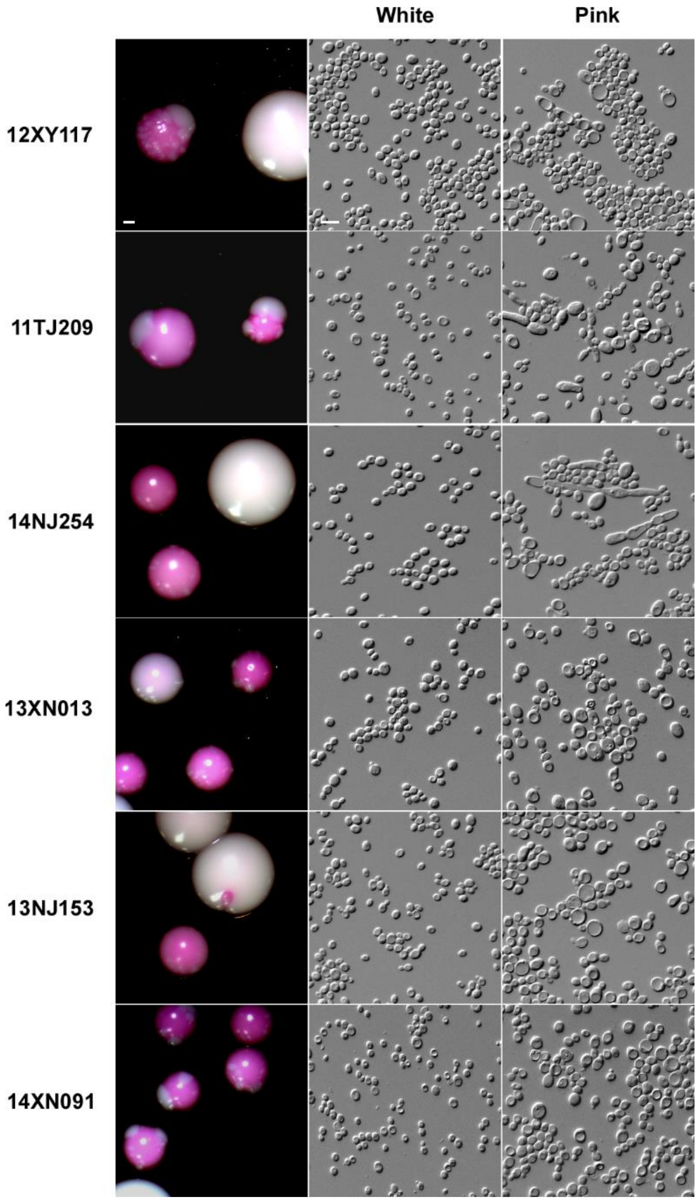

FIG S2 White-pink switching in six clinical isolates of *C. haemulonii* isolated from different hospitals in China. 12XY117 was isolated from pus culture; 11TJ209, 13XN013, and 14XN091 were recovered from venous catheter;

14NJ254, 13NJ153 were isolated from blood culture. Cells were plated on phloxine B containing YPD medium plates and incubated at 25°C for 5 days. The colony and cellular images of the white and pink phenotypes are shown. Scale bar for colonies, 1 mm; Scale bar for cells, 10 µm.

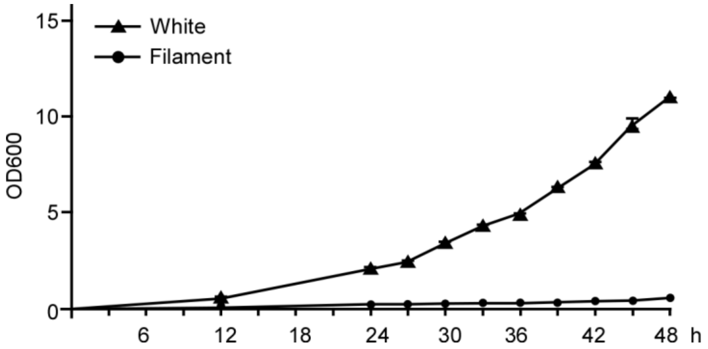

FIG S3 Growth curves of white and filament cells at 37°C. Cells from white and pink colonies on YPD agar plates were inoculated into liquid YPG medium for growth overnight at 25°C. Cells were then washed twice with double-distilled water and reinoculated into liquid YPG medium at a concentration of OD600= 0.2 and incubated at 37°C. The OD600 values were measured at different time points as indicated. Three biological repeats were performed. X-axis, incubation time; Y-axis, OD600 value.

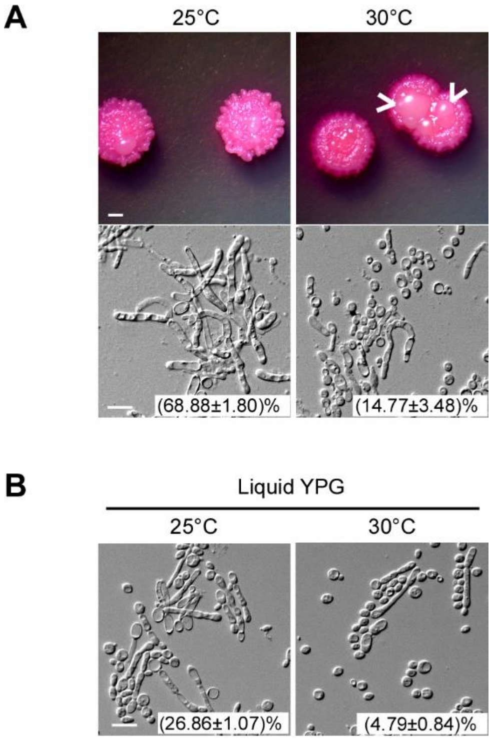

FIG S4 Effects of culture temperature on the maintenance of the filamentous phenotype. (A) Filament cells were plated on YPG media and incubated for 8 days at 25°C or 30°C. White arrows indicate the “blebs” of yeast form cells that appeared on the surface of the wrinkled colonies. (B) Filament cells (2×10^6) were inoculated in liquid YPG medium and grown for 48 h at 25°C or 30°C. Percentages of filament cells are shown in the corresponding images. Scale bar for colonies, 1 mm; scale bar for cells, 10 µm.

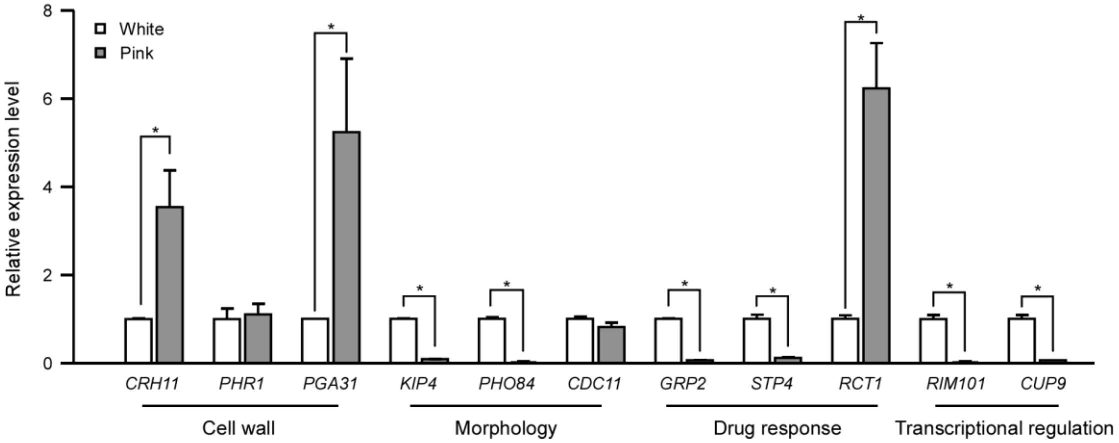

FIG S5 Relative expression levels of selected differentially expressed genes between white and pink cells of *C. haemulonii*. White and pink cells were grown in liquid YPD medium at 25°C for 36 h. Total RNA was extracted for quantitative RT-PCR assays. The relative expression levels of cell wall-associated, morphology-related, drug response and transcriptional regulatio genes were examined. The relative repression level of each gene in white cells was set as “1”. Error bars represent standard errors of three repeats. *, indicates significant difference (p<0.05, two tailed Student’s *t*-test).

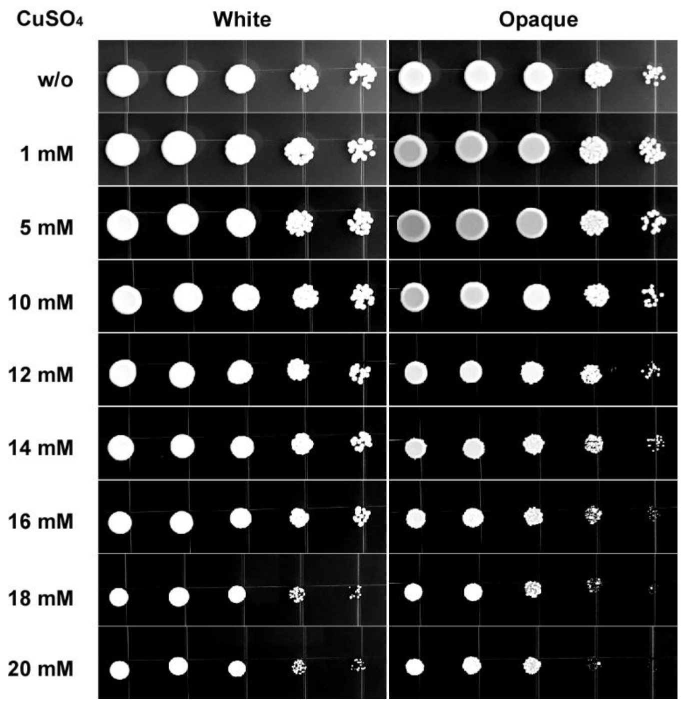

FIG S6 Inhibitory effect of $CuSO_4$ on the growth of *C. albicans* white and opaque cells at 25°C. *C. albicans* strain was adjusted to 5×10^8 cells/mL, and 10-fold serial dilutions of cells (2 µL) were spotted onto YPD and YPD agar containing serial concentrations of $CuSO_4$ for two days of growth. w/o: without $CuSO_4$.

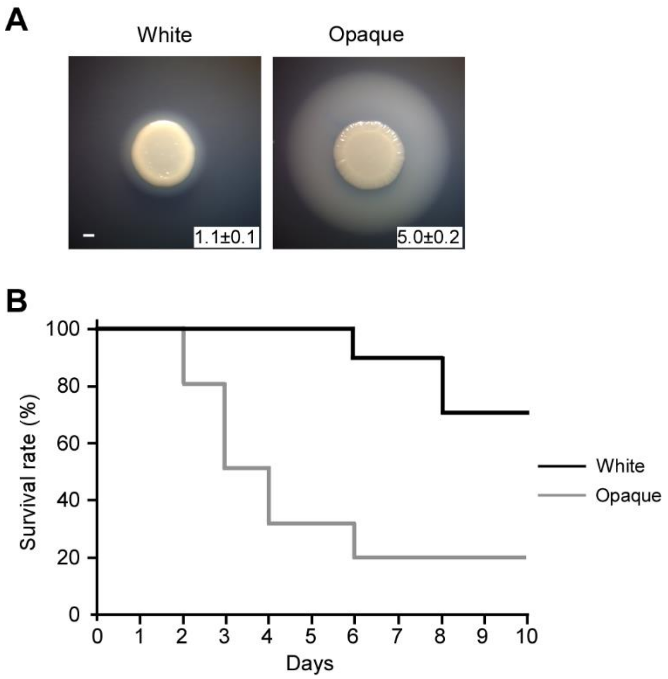

FIG S7 SAP activities of *C. albicans* white and opaque cells and virulence in a *G. mellonella* infection model. (A) SAP activity. White or opaque cells (5 × 10^6) in 5 µL double-distilled water were spotted on YCB-BSA medium and incubated at 25°C for three days. The width of the white precipitation zones representing SAP activity was measured and indicated. Cellular images of the corresponding spots are shown below. Scale bar: 1 mm (B) Survival rates of *G. mellonella* infected by white and opaque cells of *C. albicans* BJ1097 at 25°C. Cells of each cell type (1 × 10^6 cells) in 10 µL double-distilled water were injected into larvae of *G. mellonella*. For each cell type, ten larvae were used for infection.

Dataset S1. RNA-Seq analysis of white and pink cells of *C. haemulonii*. A comparative analysis of differentially expressed genes between white and pink

cells was performed. The expression profiles of all genes in the two cell types are also shown. Related to **FIG 4**.
